# Transcriptional CDK Inhibitors CYC065 and THZ1 Induce Apoptosis in Glioma Stem Cells Derived from Recurrent GBM

**DOI:** 10.3390/cells10051182

**Published:** 2021-05-12

**Authors:** Viktorija Juric, Heiko Düssmann, Martine L. M. Lamfers, Jochen H. M. Prehn, Markus Rehm, Brona M. Murphy

**Affiliations:** 1 Department of Physiology & Medical Physics, Royal College of Surgeons in Ireland, University of Medicine and Health Sciences, Dublin 2, Ireland; viktorijajuric@rcsi.com (V.J.); hduessmann@rcsi.ie (H.D.); jprehn@rcsi.ie (J.H.M.P.); 2 Centre for Systems Medicine, Royal College of Surgeons in Ireland, University of Medicine and Health Sciences, Dublin 2, Ireland; 3 Brain Tumor Center, Department of Neurosurgery, Erasmus MC, 3015 CN Rotterdam, The Netherlands; m.lamfers@erasmusmc.nl; 4 Institute of Cell Biology and Immunology, University of Stuttgart, 70569 Stuttgart, Germany; markus.morrison@izi.uni-stuttgart.de; 5 Stuttgart Research Center Systems Biology, University of Stuttgart, 70569 Stuttgart, Germany

**Keywords:** glioma stem cells, cyclin-dependent kinases, CDK inhibitors, recurrent GBM, CYC065, THZ1, CD133, CD44

## Abstract

Glioma stem cells (GSCs) are tumour initiating cells which contribute to treatment resistance, temozolomide (TMZ) chemotherapy and radiotherapy, in glioblastoma (GBM), the most aggressive adult brain tumour. A major contributor to the uncontrolled tumour cell proliferation in GBM is the hyper activation of cyclin-dependent kinases (CDKs). Due to resistance to standard of care, GBMs relapse in almost all patients. Targeting GSCs using transcriptional CDK inhibitors, CYC065 and THZ1 is a potential novel treatment to prevent relapse of the tumour. TCGA-GBM data analysis has shown that the GSC markers, CD133 and CD44 were significantly upregulated in GBM patient tumours compared to non-tumour tissue. CD133 and CD44 stem cell markers were also expressed in gliomaspheres derived from recurrent GBM tumours. Light Sheet Florescence Microscopy (LSFM) further revealed heterogeneous expression of these GSC markers in gliomaspheres. Gliomaspheres from recurrent tumours were highly sensitive to transcriptional CDK inhibitors, CYC065 and THZ1 and underwent apoptosis while being resistant to TMZ. Apoptotic cell death in GSC subpopulations and non-stem tumour cells resulted in sphere disruption. Collectively, our study highlights the potential of these novel CKIs to induce cell death in GSCs from recurrent tumours, warranting further clinical investigation.

## 1. Introduction

Gliomas represent the most common primary malignancy of the central nervous system (CNS) and grade IV glioma (glioblastoma (GBM)) are the most aggressive ones. Currently, GBM standard of care (SOC) includes surgery followed by radiotherapy (RT) and temozolomide (TMZ) chemotherapy [1]. Virtually all GBM patients suffer from tumour relapse and no effective or common established SOC exists for patients with recurrent GBM. Due to lack of effective treatment options, especially in the recurrent setting, GBM is generally lethal, with median survival being less than 15 months.

Cancer stem cells (CSCs) are defined as subpopulations of cells within a tumour that are capable of aberrant differentiation, self-renewal and tumour initiation. Resistance to radio- and chemotherapy in GBM is understood to arise from glioma stem cells (GSCs) [2,3]. GSCs employ numerous strategies to resist radiotherapy and cytotoxic chemotherapeutics, for example by the expression of several ABC transporters, resistance to programmed cell death and increased DNA repair capability [4]. Additionally, growing evidence also suggests the GSC microenvironment is involved in mediating GBM radiation-resistance [5]. Several markers have been used so far in order to identify stem-like cells in GBM although, no markers have been identified that exclusively and comprehensively mark GSCs [6]. The most commonly used is a cell surface marker, CD133—a transmembrane glycoprotein consisting of five transmembrane domains. When first identified in GBM, CD133 positive cells were shown to be highly proliferative and capable of initiating tumour growth in NOD/SCID mouse brains whilst CD133-negative cells did not [7]. Similarly, CD133-negative cells showed no ability to form spheres when separated from the CD133-positive subpopulations [8,9]. On the other hand, more recent studies have highlighted the existence of both CD133-positive and -negative subpopulations in tumour initiating cells [10]. CD133 expression has also been shown to negatively correlate with GBM patient survival [11] and can be used as a prognostic marker. CD44 is another cell surface marker frequently used in GBM studies as well as other tumour types [12,13] and is associated with tumour invasion and aggressive growth of GBM tumours [14,15]. It has also been shown that a higher CD44 expression in GBM patients correlates with worse patient prognosis due to increased tumour cell proliferation, invasion and resistance to radio- and chemotherapy [16]. 

CDKs are critical regulatory enzymes that drive all cell cycle transitions [17]. CDK1, -2, -4 and -6, directly promote cell cycle progression, while CDKs, such as CDK7, -8 and -9 regulate transcription [18]. As one of the most fundamental traits of cancer cells involves their ability to sustain chronic proliferation, it is unsurprising that virtually all cancers, including GBM, harbour genomic alterations that lead to the constitutive activation of CDKs, resulting in unchecked cancer cell growth and division. Such observations have resulted in the expansion of translational research in the CDK inhibitor space, in particular for those tumours that are resistant to established treatments. Here, we examined the ability of two transcriptional CDK inhibitors, CYC065 and THZ1 to target recurrent GBM cultured as gliomaspheres. CYC065 is a second generation CDK inhibitor, that primarily targets CDK9 and in comparison to its first generation compound, R-roscovitine has significantly improved metabolic stability, efficacy and potency in vitro and in vivo [19,20]. It has been shown to reduce tumour growth in an orthotopic mouse model of GBM and can cross the blood–brain barrier (BBB) [19]. On the other hand, THZ1 specifically targets CDK7 and induces apoptotic cell death in preclinical studies of blood and solid tumours [21,22,23,24,25]. While THZ1 has not yet reached clinical trials, CYC065 is in early phase trials for blood (NCT04017546, NCT03739554) and solid tumours (NCT02552953) and shown to be well tolerated in patients. In order to examine the clinical utility of these CKIs for recurrent GBM, we examined their ability to induce the death of patient-derived GSC enriched gliomasphere cultures from recurrent tumours.

## 2. Materials and Methods

### 2.1. Cell Lines and Cell Culture

Two recurrent patient-derived tumour cultures (GTCC-9, GTCC-10) were generated from the GBM tissue samples provided by the Department of Neurosurgery of the ErasmusMC (Rotterdam, The Netherlands), and obtained as part of routine resections from patients under their informed consent (ethical approval number MEC-2013-090). Patient clinical characteristics are given in Table 1.

GBM cultures were used up to passage number 25 and grown in freshly prepared (every two weeks) DMEM-F12 medium containing B27 supplement 50X (2%) (Gibco Life Technologies, Dún Laoghaire, Ireland), human bFGF (20 ng/mL), human EGF (20 ng/mL) (PeproTech EC Ltd., London, UK), penicillin/streptomycin (100 U/mL) (Sigma-Aldrich, Arklow, Ireland), heparin (5 µg/mL, Alfa Aesar, Heysham, UK). Cells were grown as gliomaspheres in non-coated plates or ECM (1:100, ECM, Cultrex, PathClear, Trevigen, MD, USA) coated flasks as a monolayer cultures and maintained in a humidified incubator at 37 °C and 5% CO_2_. Cells were routinely tested for mycoplasma infection and were mycoplasma free.

### 2.2. Stem Cell Population Characterisation 

GBM cells were cultured as gliomaspheres and left untreated for basal expression analysis or treated with 150 μM TMZ (#T2577, Sigma-Aldrich, Arklow, Ireland), 3 μM CYC065 (#HY-101212, Medchemexpress, NJ, USA) or 100 nM THZ1 (#HY-80013, Medchemexpress, NJ, USA) for 120 h. At the end of treatment window, cells were harvested, dissociated using Accutase (Thermo Fisher Scientific, Waltham, MA, USA) and washed in FACS washing media (DPBS + 5% FCS, Gibco Life Technologies, Dún Laoghaire, Ireland). Cells were incubated for 15 min in FACS wash media on ice and stained with staining solution: unstained control, CD133-VioBright 667 (#130-111-756, MACS, Miltenyi Biotec, Bisley, UK), VioBright 667-isotype control (#130-118-217, MACS, Miltenyi Biotec, Bisley, UK), CD44-FITC (#555478, BD Pharmingen, Franklin Lakes, NJ, USA), FITC-isotype control (#555748, BD Pharmingen, Franklin Lakes, NJ, USA) for 30 min on ice, protected from light. All antibodies were diluted as recommended by suppliers. VioBright 667 was excited at 638 nm and fluorescence emission was collected through a 660 nm long pass filter. FITC was excited at 488 nm and fluorescence emission was collected in the FL1 channel through a 520 nm band-pass filter. A total of 1 × 10^4^ gated cells were acquired. Isotype controls were used to set CD133-positive and CD44-postive gates. FACS analysis was done using Attune NtX flow cytometer and data was analysed using FlowJo Software 10.6.2. Version 5.0 (Becton, Dickinson and Company, Franklin Lakes, NJ, USA).

### 2.3. Western Blot Analysis

Whole-cell lysates were prepared using RIPA lysis buffer containing 150 mM NaCl, 0.1% Triton X-100, 0.5% sodium deoxycholat, 0.1% sodium dodecyl sulfate (SDS), 50 mM Tris in ddH_2_O, pH 8, and protease/phosphatase inhibitor cocktails (Sigma-Aldrich, Arklow, Ireland). BCA protein assay kit (Pierce, Rockford, IL, USA) was used to determine the protein concentrations in the cell lysates. An equal amount of lysates (20 μg) were diluted with Laemmli loading buffer and were separated on 12% SDS-polyacrylamide gels and transferred to nitrocellulose membranes using wet transfer. Membranes were blotted with primary antibodies at the following dilutions: Cdk2 (#sc-6248, 1:500), Cdk7 (#sc-7344, 1:500), Cdk9 (#sc-13130, 1:500) (Santa Cruz Biotechnology, Santa Cruz, CA, USA), cleaved caspase-3 (#9661, 1:1000), cleaved caspase-7 (#9491, 1:1000) (Cell Signaling, Danvers, MA, USA), α-tubulin (#T-6195, 1:5000) and GAPDH (#MAB374, 1:5000) (Sigma-Aldrich, Arklow, Ireland). Membranes were next incubated with mouse (#AP124P, 1:5000) or rabbit (#AP132P, 1:5000) (Merck KGaA, Darmstadt, Germany) horseradish peroxidase-conjugated secondary antibodies and protein bands were visualised using Supersignal West Pico Chemiluminescent Substrate (Pierce). Images were captured using Fuji-film LAS-4000 (Fuji, Sheffield, UK).

### 2.4. Cell Death Analysis Using Flow Cytometry

Cell death was measured using a BD LSRII flow cytometer. Recurrent GBM cells were seeded as gliomaspheres, 300,000 cells per well in 6-well plates. After 24 h cells were either pretreated with 150 μM TMZ for 24 h followed by treatment with 3 μM CYC065 or 100 nM THZ1 for 120 h or treated with 3 μM CYC065 and 100 nM THZ1 as single agents for 120 h. Following treatments, gliomaspheres were pelleted, dissociated using Accutase and were next incubated in 100 μL of binding buffer (10 nM HEPES, 135 nM NaCl, 5 mM CaCl_2_) containing AnnexinV-FITC conjugate (1:200, BioVision, Mountain View, CA, USA) and propidium iodide (1:500, Sigma-Aldrich, Arklow, Ireland) for 10 min on ice in the dark. FITC was excited at 488 nm and fluorescence emission was collected in the FL1 channel through a 520 nm band-pass filter. PI was excited at 561 nm and fluorescence emission was collected through a 605/40 nm band-pass filter and a 570 nm long pass filter. A total of 1 × 10^4^ gated cells were acquired. Acquired data from the flow cytometry analyses were analysed using FlowJo Software.

### 2.5. siRNA Transfection

To determine the effect of CDK2, CDK9 and CDK7 silencing on cell viability/death and morphology in GBM recurrent cultures, siRNA targeting the CDK2, CDK9 or CDK7 gene was transiently transfected into GTCC-9 and GTCC-10 cell lines using Lipofectamine 2000 transfection reagent (Thermo Fisher Scientific, Waltham, MA, USA) according to the manufacturer instructions. Briefly, cells were seeded as 2-D cultures in 6-well plates (300,000 cells/well) coated with extracellular matrix (1:100) and transfected with 20 nM of CDK2, CDK9 or CDK7 siRNA or 20 nM control siRNA (Thermo Fisher Scientific, Waltham, MA, USA) in Opti-MEM (Gibco Life Technologies, Dún Laoghaire, Ireland). Twenty-four hours post transfection cells were detached and plated in DMEM-F12 complete medium in non-coated plates as described above. Whole cell lysates were collected 24 h post re-plating as described previously and transfection efficacy was determined using Western blot analysis. Flow cytometry and fluorometric cell viability were conducted 48 h after siRNA transfections as described.

### 2.6. Fluorometric Cell Viability and Cytotoxicity Detection

After indicated genetic depletions cells were seeded as 3000 cells/well in 96-well plate. Following 24 h incubation gliomaspheres were stained with Calcein AM (Invitrogen, Waltham, MA, USA). 4 μM Calcein AM in DPBS was added to a 15 mL falcon tube, mixed and incubated at 37 °C for 15 min. Media was then removed from the cultures and 100 µL of Calcein AM/DPBS mix was added/well for 30 min at 37 °C prior to imaging. Images were taken immediately with an Eclipse TE300 inverted microscope using FITC channel.

### 2.7. Fluorescence-Activated Cell Sorting (FACS)

For flow cytometry sorting and analysis, cells were dissociated with Accutase and labelled with CD44-FITC and CD133-VioBright 667 along with the relevant isotype controls for gating. Gating for single cells was established using forward scatter in the isotype control sample. The isotype control sample was used to establish a gate in the FITC and VioBright 667 channel. Cells showing signal for CD133 above the gate established by the isotype control were deemed to be CD133-positive cells. Cells showing signal for CD44 above the gate established by the isotype control were deemed to be CD44-positive cells (Figure S1). BD FACSAria III flow cytometer was used for the analysis. Upon sorting cells were cultured in complete DMEM-F12 complete medium and spheres were allowed to reform before further experiments were undertaken.

### 2.8. Light Sheet Fluorescence Microscopy (LSFM)

Gliomaspheres were seeded as 5000 cells/well in 96 well plate, pre-stained with 1 μg/mL Hoechst 33258 (Sigma-Aldrich, Arklow, Ireland). Cells were grown overnight at 37 °C in 5% CO_2_. For imaging, the spheroids were embedded in 1% low-melting agarose in DPBS (Sigma Aldrich, Arklow, Ireland) at 38–40 °C doped with sub-resolution beads at a concentration of 1:1000 of the original stock (PS-Speck, Thermo Fisher, Ireland), sucked into a glass capillary while liquid. Spheres were then stained as per protocol described above using in house made holder to enable immersion of the hardened agar with the sphere into the prepared staining solution (1:10 CD133-VioBright 667 and 1:100 CD44-FITC) on ice as per manufacturer’s instructions. For imaging the capillary was mounted in the microscope sample holder and the chamber of the Light Sheet Fluorescence Microscope (Lightsheet Z1, Carl Zeiss, Germany). A plunger was used to push the agar with the embedded spheroids out of the capillary into the liquid in front of the 20 × 1.0 NA lens. The light sheet was generated with two 10 × 0.2 NA lenses illuminating the sample alternating from each side using the pivot scan mode. Images were taken at zoom 1.0 resulting in a light sheet thickness of 3.3 µm (405 nm excitation). Hoechst 33258 was excited using the 405-nm laser line, VioBright 667 with 639 nm and FITC with 488 nm all using the 405/488/561/640-nm notch filter. To split the emission onto the two PCO edge sCMOS cameras filter cubes with beam splitter 510 nm using band-pass filters of 420–470 nm (Hoechst 33258), LP 660 nm (VioBright 667) and 505–545 nm (FITC). Image stacks were then taken moving the object along the optical axis of the imaging objective at 1 µm steps and subsequently turning the object in 45° steps and re-aligning the object for each of the next 4 stacks. Stacks in Figure 2A where taken at zoom 2 with 2.91 μm average light sheet thickness at the sample and 0.114 μm represented by one pixel size, stacks in Figure 2B were taken at zoom 1.2 with 3.7 μm average light sheet thickness at the sample and 0.19 μm represented by one pixel. Stacks in Figure 3 were taken with zoom 1, average 3.25 μm light sheet thickness and 0.228 μm represented by one pixel. All images were processed using ZEN black and FiJi (ImageJ 1.52r-t). The multi-view fusion and deconvolution were performed using the dedicated FiJi plugins (MultiView Reconstruction v5.0.2034) [26] using the sub-resolution beads present in the agarose as an alignment aid for registration and for the generation of the PSF used for deconvolution. For the image fusion processing, the scale was set to half of the original resolution in Figure 2 while it was processed 1:1 for Figure 3 using a server with dual Xeon Gold and 192 GB RAM (Power Edge R 740 XD, Dell EMC), deconvolution was done with 512^3^-pixel block size. Processed stacks are then presented in the figures as indicated.

### 2.9. Laser Scanning Confocal Microscopy

Recurrent cultures were cultured as 2-D or 3-D (as described above). Cell were pre-stained with Hoechst 33258 (1 μg/mL, nuclei staining). On the day of imaging 2-D cultures and gliomaspheres were stained with anti-CD133 and anti-CD44 antibodies (as per manufacturer’s instructions, described above). Samples were then mounted on an LSM 710 confocal laser-scanning microscope (Carl Zeiss, Germany) fitted with a 40×/1.4 NA Plan-Apochromat oil immersion objective. Lasers of sizes 405 nm, 488 nm and 633 nm were used to excite Hoechst 33258, CD44-FITC and CD133-VioBright 667 conjugated antibodies with detection ranges of 415–494 nm, 490–544 nm and 638–728 nm respectively. Further image processing (background subtraction, median filtering and was carried out using Fiji/ImageJ (version 1.52i, Wayne Rasband, NIH)).

### 2.10. Fluorescence Imaging Using Nikon Eclipse TE2000 Microscope

Cells were pre-stained with Hoechst 33258 (1 μg/mL). Prior to imaging cells were stained with AnnexinV-FITC (1:200) and PI (1:500) whilst mitochondrial potential was analysed by Mitotracker Deep Red (30 nM final concentration, Biosciences, Ireland) uptake. CaCl_2_ (20 µM final concentration) was added to facilitate AnnexinV binding. Images were taken using a Nikon TE2000 fluorescence microscope, 20×/0.45 NA objective and Orca 285 CCD camera (Hamamatsu, Molecular Devices, San Jose, CA, USA) with single bandpass, high transmission filtersets for all four fluorescent stains (Semrock and Chroma, AHF, Tübingen, Germany).

### 2.11. TCGA Data Analysis

Tumour gene expression and patient information were obtained from publicly available database, The Cancer Genome Atlas (TCGA, https://www.cancer.gov/tcga, accessed on 21 August 2018) [27]. TCGA dataset was accessed through open-access GlioVis portal [28].

### 2.12. Statistics and Software

All data from Western blotting were representative of at least three independent experiments. Statistical analyses were performed using GraphPad Prism software version 8.4.3 (GraphPad software Inc., La Jolla, CA, USA). Results are presented as mean ± SEM. Data were tested for significance using appropriate tests as detailed in the respective figure legends (* denotes *p* values < 0.05, ** denotes *p* values < 0.01, *** denotes *p* values < 0.001, **** denotes *p* values < 0.0001 and were considered to be statistically significant; ns = not significant).

## 3. Results

### 3.1. Expression of GSCs Markers, CD133 and CD44 Is Upregulated in GBM

**Figure 1:**
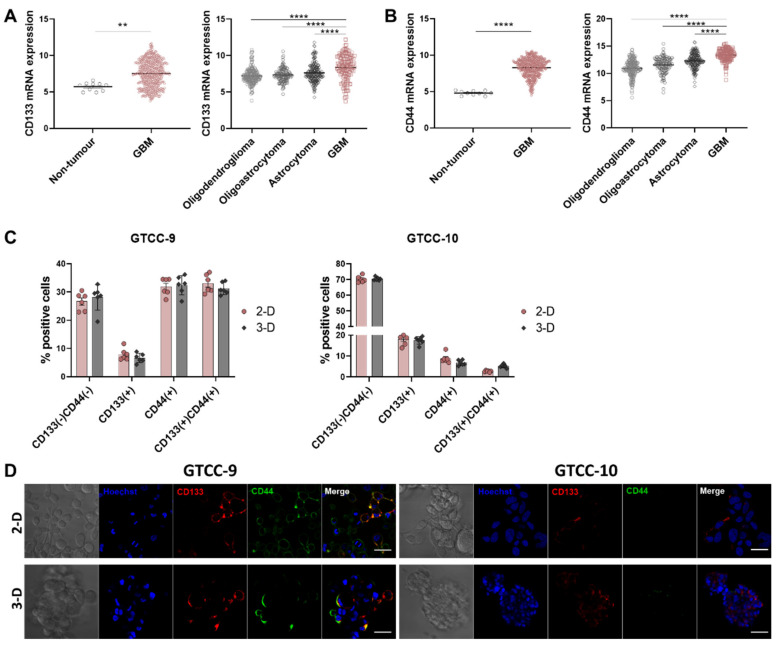
CD133 and CD44 GSC biomarkers are upregulated in GBM. (**A**) Expression of CD133 stem cell marker in the TCGA-GBM dataset. Two-tailed unpaired *t*-test was used to test for significance, whereby ** *p* < 0.01 (left panel). Expression of CD133 stem cell marker in the TCGA-LGG-GBM dataset. Two-way ANOVA with post-hoc Tukey’s analysis was used for statistical analysis, whereby, **** *p* < 0.0001 (right panel). (**B**) Expression of CD44 stem cell marker in the TCGA-GBM dataset. Two-tailed unpaired t-test was used to test for significance, whereby **** *p* < 0.0001 (left panel). Expression of CD44 stem cell marker in the TCGA-LGG-GBM dataset. Two-way ANOVA with post-hoc Tukey’s analysis was used for statistical analysis, whereby, **** *p* < 0.0001 (right panel). (**C**) CD133 and CD44 expression in recurrent monolayer (2-D) and gliomasphere (3-D) cultures was assessed using flow cytometry. Data are expressed as mean ± SEM. *n* = 6 independent experiments performed in triplicate. (**D**) Confocal microscopy was used to assess the expression of CD133 and CD44 stem cell markers in GTCC-9 (left) and GTCC-10 (right) cultures in 2-D and 3-D culturing conditions; representative images are shown here; scale bar = 50 μm.

One of the key features contributing to GBM tumour recurrence is proposed to be an underlying subpopulation of GSCs that remain resistant to SOC [29]. We selected two commonly used and well characterised glioma stem cell markers, CD133 and CD44 in order to identify GSC subpopulations for our study [30]. Analysis of the TCGA-GBM dataset showed significant upregulation of CD133 and CD44 stem cell surface markers in GBM tumour tissue compared to non-tumour tissue (Figure 1A,B, left panel), in agreement with previous studies [27]. We interrogated tumours of differing grade and observed a higher expression of CD133 and CD44 in glioblastoma compared to lower grade gliomas (Figure 1A,B, right panel) in TCGA-LGG-GBM dataset. Of note, CD133 was most prevalent in the proneural subtype whilst CD44 expression was highest in the mesenchymal subtype of GBM (Figure S2). In order to assess new treatment options for recurrent GBM, we established two gliomasphere cultures derived from patients with recurrent GBM. Flow cytometry analysis and fluorescence imaging were conducted using anti-CD133 and anti-CD44 antibodies and we observed a substantial expression of CD133 positive GSCs in both GTCC-9 and GTCC-10 gliomasphere cultures whilst CD44 positive GSCs were largely restricted to GTCC-9 gliomaspheres (Figure 1C,D). Of note, the levels of GSC biomarkers in monolayer cultures were similar to those in 3-D gliomasphere cultures (Figure 1C,D).

**Figure 2:**
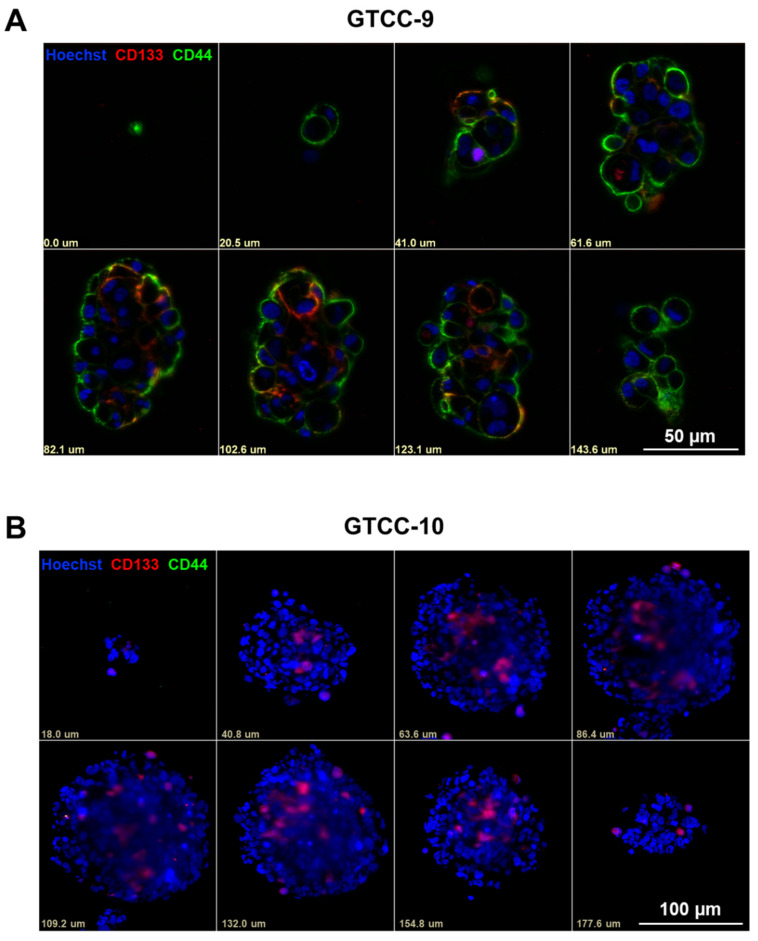
Localisation of CD133 and CD44 stem cell markers within recurrent gliomaspheres. (**A**,**B**) Light-sheet fluorescence microscopy was used to assess the localisation of CD133 and CD44 stem cell biomarkers in GTCC-9 (**A**) and GTCC-10 (**B**) gliomaspheres; representative images are shown here; scale bar = 50 μm (**A**); scale bar = 100 μm (**B**); steps between optical slices indicated in µm in (**A**,**B**); *n* = 3 independent experiments performed.

Next we determined the localisation of the specific stem cell markers within the gliomaspheres using Light Sheet Fluorescence Microscopy (LSFM). In the GTCC-9 gliomasphere cultures we observed that the CD133 biomarker was expressed on the cells within the core of the spheres, while the CD44 biomarker was more ubiquitously expressed and was evident on the cells localized both internally and externally in the spheres (Figure 2A). We already observed that cells in the GTCC-10 gliomaspheres more frequently expressed the CD133 biomarker compared to the CD44 biomarker (Figure 1C,D), and this was also borne out in the LSFM imaging (Figure 2B). The CD133 biomarker was found to be expressed on the cells in the core of the spheres while no clear conclusions could be made regarding the CD44 biomarker localisation within the spheres (Figure 2B). These results highlight that recurrent gliomaspheres express stem cell biomarkers in a heterogeneous manner which is a reflection of the intratumour heterogeneity found in GBM [31].

### 3.2. CD133 and CD44 GSC Biomarker-Negative Cells Convert into GSC Biomarker-Positive Cells and Contribute to the Enrichment in GSCs in Recurrent GBM 

**Figure 3:**
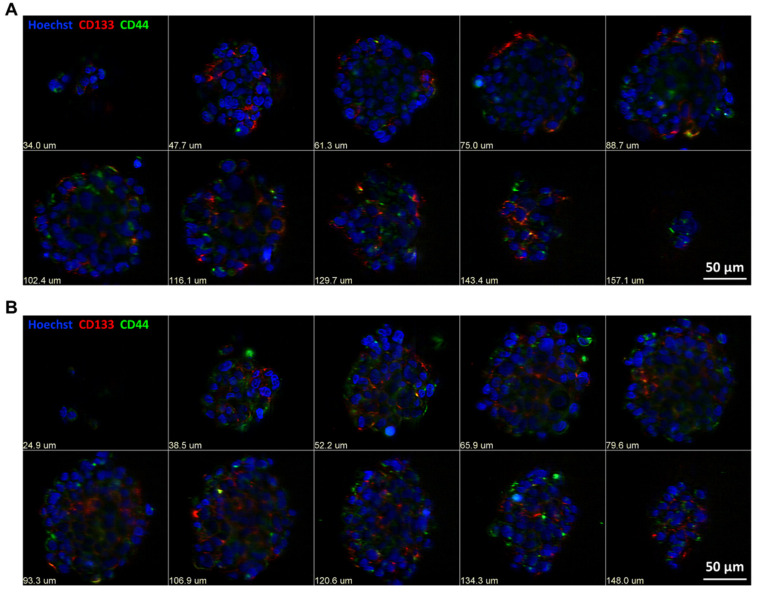
Subsets of CD133 and CD44 biomarker-negative cells convert into biomarker-positive GSC cells. (**A,B**) Light Sheet Florescence Microscopy (LSFM) was used to assess the expression of CD133 and CD44 positive cells in GTCC-10 gliomaspheres; representative images are shown here; scale bar = 50 μm; steps between optical slices indicated in µm; *n* = 3 independent experiments performed in triplicate.

While the above results supports that culturing patient-derived cells in serum-free media contributed to GSC biomarker maintenance, we were now interested to interrogate if GSC biomarker-negative cells can convert into GSC biomarker-positive cells. To assess this, FACS was performed to obtain CD133 and CD44 biomarker-negative cell populations of the GTCC-10 cell line (Figure S1), which were then cultured to assess the plasticity of the GSC biomarker-negative cells. Twelve days in culture resulted in enrichment for both CD133 and CD44 biomarker-positive cells (Figure 3A,B). Both CD133 and CD44 positive cells were very prominent in the outer layers and core of the spheres (Figure 3A,B).

### 3.3. CDK2/9-Targeting, CYC065 and CDK7-Targeting, THZ1 Induce Apoptotic Cell Death in Recurrent Gliomaspheres 

**Figure 4:**
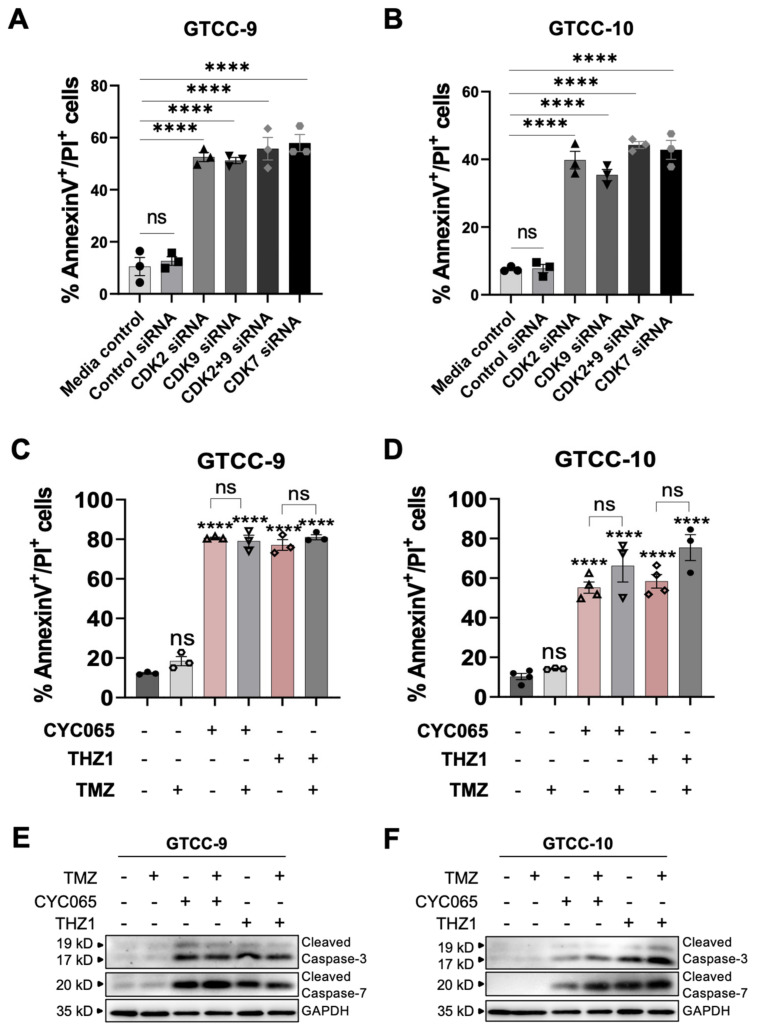
Recurrent gliomaspheres are resistant to TMZ but sensitive to CYC065 and THZ1 as single agents. (**A**,**B**) Apoptotic cell death was measured using AnnexinV/PI staining in cells transfected with scrambled control siRNA (20 nM), Cdk2 and/or 9, 7-targeting siRNAs (20 nM) (as indicated) in GTCC-9 (**A**) and GTCC-10 (**B**) cultures. Data are expressed as mean ± SEM. One-way ANOVA with post-hoc Tukey’s analysis was used for statistical analysis, whereby, **** *p* < 0.0001, ns = not significant; *n* = 3 independent experiments performed. (**C**,**D**) Apoptotic cell death upon treatment with DMSO, 150 μM TMZ, 3 μM CYC065 or 100 nM THZ1 alone or pre-treated with TMZ (150 µM, 24 h) was determined using flow cytometry AnnexinV/PI staining 120 h post treatment. Data are expressed as mean ± SEM. One-way ANOVA with post-hoc Tukey analysis was used for statistical analysis, whereby, **** *p* < 0.0001, ns = not significant; *n* = 3 independent experiments performed in triplicate for each condition. (**E**,**F**) Caspase activation was followed using Western blot analysis in two recurrent cell lines treated for 120 h with DMSO, 150 μM TMZ, 3 μM CYC065 or 100 nM THZ1 alone or pre-treated with TMZ (150 µM, 24 h). GAPDH was used as a loading control. Western blot analysis was performed for *n* = 3 biological replicates and representative blots are shown here.

The essential roles of CDKs in the intracellular control of the cell cycle, regulation of transcription and DNA repair make them highly suitable as targets of inhibitors for the treatment of cancer. We have previously demonstrated that inhibiting CDKs 2 and 9 induce apoptotic cell death in vitro and in vivo [19]. We now wanted to examine the efficacy of CKIs to target recurrent GBM specifically. Due to upregulation of CDK2, 7 and 9 in GBM [28] we first examined effects of genetic depletion of CDK2, 7 and 9 in recurrent gliomaspheres. Upon successful depletion of the CDKs in the gliomaspheres (Figure S3A), morphological differences between control treated and specific gene depleted cells were observed using bright field microscopy. Genetic depletion of CDK2/9 and 7 significantly decreased the size and viability of both gliomaspheres as observed using Calcein staining (Figure S3B,C). Additionally, genetic depletion of CDK2 and/or 9 and 7 induced significant levels of apoptotic cell death in GTCC-9 and GTCC-10 gliomaspheres compared to the control (Figure 4A,B). In order to translate previous findings into more clinically applicable settings, we next applied specific CDK2/9 (CYC065) and CDK7 (THZ1) inhibitors as single treatments and in combination with conventional chemotherapy, TMZ. Our study demonstrated that 120 h post treatment CYC065 and THZ1 induced significant levels of apoptotic cell death while TMZ was ineffective as a single agent (Figure 4C,D). Interestingly, CYC065 and THZ1 induced higher levels of apoptotic cell death when pretreated with TMZ in GTCC-10 gliomaspheres (Figure 4D). While the observed changes were not statistically significant this supports the potential of these treatments in recurrent GBM, previously treated with TMZ as frontline therapy. Western blot analysis showed activation of executioner caspases-3 and -7 in the CYC065 and THZ1 treated groups while activation of caspases was not observed in control and TMZ-single agent treated groups (Figure 4E,F). Collectively, this data demonstrated that pharmacological inhibition of CDK2/9 or 7, using CYC065 and THZ1, respectively induce apoptotic cell death in recurrent gliomaspheres which are rich in GSCs and are highly resistant to TMZ.

### 3.4. CYC065 and THZ1 Decrease the Expression of the CD133 Stem Cell Marker in Recurrent Gliomaspheres and Induce Apoptotic Cell Death in the Glioma Stem Cell Subpopulations

**Figure 5:**
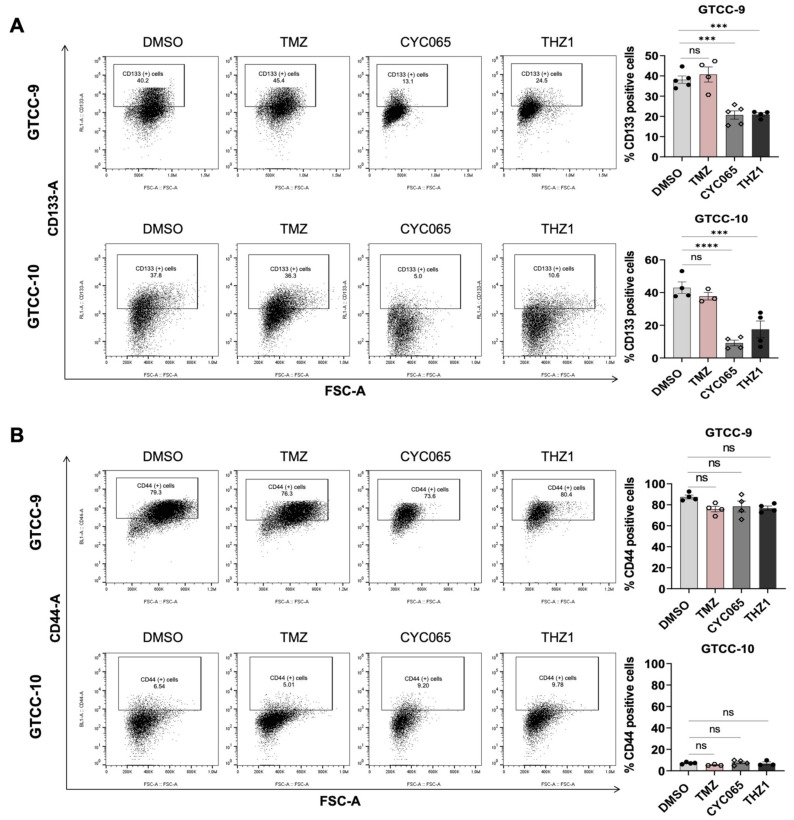
CYC065 and THZ1 decrease the expression of CD133 stem cell marker in recurrent gliomaspheres. (**A**,**B**) Flow cytometry was used to assess the expression of CD133 and CD44 after 72 h treatment with 150 μM TMZ, 3 μM CYC065 or 100 nM THZ1 in GTCC-9 and GTCC-10 recurrent gliomaspheres. Representative plots and quantification of at least three experiments are shown here. Data are expressed as mean ± SEM. One-way ANOVA with post-hoc Tukey’s analysis was used for statistical analysis, whereby, *** *p* < 0.001, **** *p* < 0.0001, ns = not significant; gate settings—Figure S4.

To assess whether the expression of CD133 and CD44 stem cell markers alter upon treatment with the CKIs and TMZ, flow cytometry analysis was conducted in the two recurrent gliomasphere cultures. CYC065 and THZ1 significantly decreased the expression of the CD133 stem cell marker in both cultures (Figure 5A) while neither treatment notably changed CD44 stem cell marker expression (Figure 5B). On the other hand, TMZ did not significantly affect the expression of either marker in the gliomasphere cultures (Figure 5A,B).

**Figure 6:**
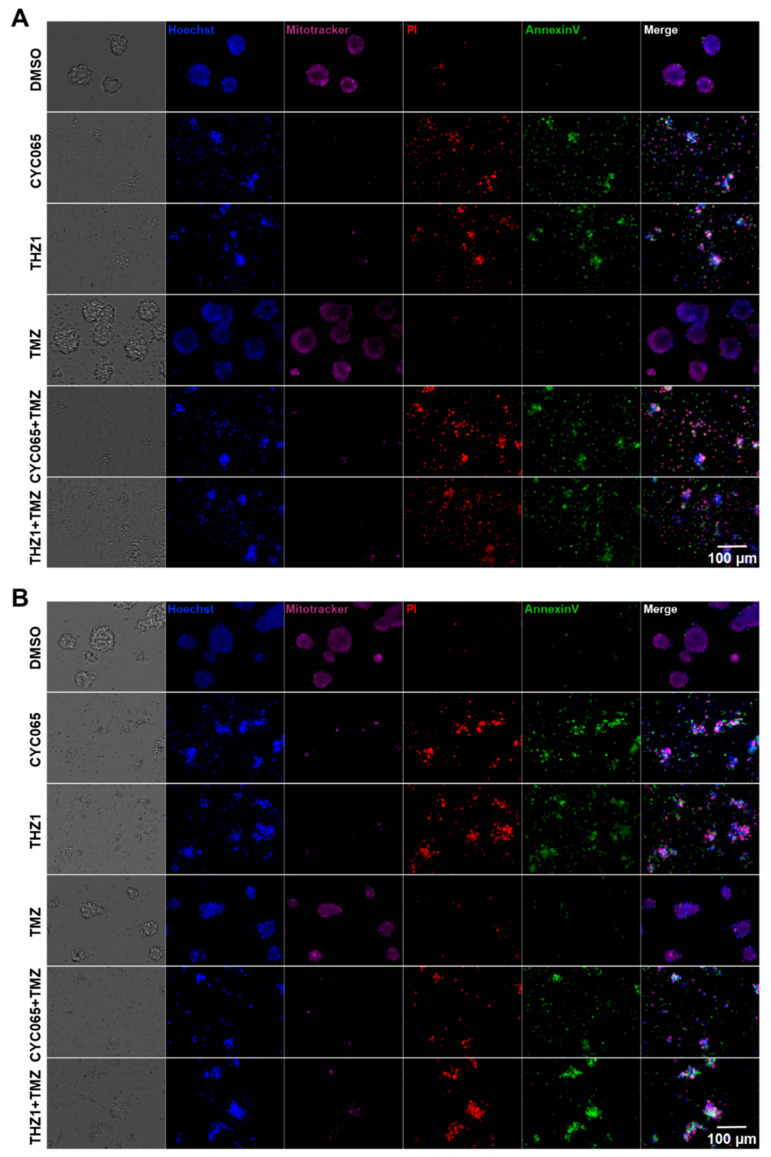
CYC065 and THZ1 induce apoptotic cell death in CD133 and CD44-positive GSC subpopulations in recurrent GTCC-10 gliomaspheres. (**A**) CD133-positive subpopulation of GTCC-10 gliomaspheres and (**B**) CD44-positive subpopulation of GTCC-10 gliomaspheres. Sorted subpopulations were treated with DMSO, 150 μM TMZ, 3 μM CYC065 or 100 nM THZ1 alone or pre-treated with TMZ (150 µM, 24 h) and imaged 120 h after treatment. Images were taken using Nikon Eclipse TE2000 microscope; scale bar = 100 μm; *n* = 3 independent experiments were performed.

In order to further interrogate glioma stem-cell treatment susceptibility we utilised FACS to isolate four cell subpopulations—CD133-single positive, CD44-single positive, CD133-CD44-double positive and CD133-CD44-double negative (Figure S1). These subpopulations were then treated with CKIs and/or TMZ and subsequently imaged. We observed that CYC065 and THZ1 disrupted sphere formation and induced depolarisation of the mitochondrial membrane in CD133-positive GSC subpopulations (Figure 6A) and CD44-positive GSC subpopulations (Figure 6B) (bright field and Mitotracker Deep Red). Additionally, CKIs as single agents also induced apoptosis in both CD133-positive (Figure 6A) and CD44-positive subpopulations of GTCC-10 GSCs (Figure 6B) (PI and AnnexinV positive). On the other hand, TMZ alone did not disrupt sphere formation nor did it induce significant levels of apoptotic cell death in either of the stem cell subpopulations. Similar results were obtained in CD133-CD44 GSC double biomarker-negative subpopulations and CD133-CD44 GSC double biomarker-positive subpopulations in GTCC-10 gliomaspheres—reduction in sphere size, depolarization of the mitochondrial membrane followed by an increase in PI uptake and AnnexinV staining (Figure S5A,B). These data demonstrate that CYC065 and THZ1 induce apoptotic cell death in CD133 and CD44 GSC biomarker-positive subpopulations in recurrent GSCs where TMZ has little or no effect. Furthermore these inhibitors are also effective against biomarker-negative subpopulations, an important attribute especially considering the plasticity of the GSCs (Figure S5A). Collectively, these results highlight the potential of CKIs as an alternative to conventional treatment to overcome treatment resistance in recurrent GBM. 

## 4. Discussion

GBM is the most common and aggressive form of brain tumour, with a dismal therapeutic outcome for the majority of patients. Despite radiotherapy and adjuvant chemotherapy, patient survival still remains low due to tumour recurrence [32]. Depending on a patient’s physical condition, once a tumour recurs they either undergo RT and chemotherapy or when possible surgery followed by RT and chemotherapy [32]. Currently there is no SOC for recurrent GBM. Thus, identification of novel molecular targets is critical for the development of successful therapies for this highly aggressive and deadly type of brain tumour. In the last decade CKIs have been studied as a potential novel treatment in many cancers including GBM [18]. The CDK2, 7 and 9 genes are frequently upregulated in GBM patients compared to non-tumour tissue [27] and thus establishes a therapeutic rationale for targeting these CDKs specifically, using transcriptional CDK inhibitors, CYC065 (CDK2/9i) and THZ1 (CDK7i).

Growing evidence suggest that cancer stem cells (CSCs) play a critical role in tumour progression, metastasis and drug resistance. Targeting resistant subpopulations of CSCs in GBM has been attempted in the past using drugs against PKCε, AKT and XIAP [33], PTEN/PI3K/Akt [34] and Hedgehog/Gli [35] pathway but have not yet progressed into clinical trial. Even though many studies of CSCs in GBM have been undertaken, data showing which specific markers should be used are still contradictory. CD133 is the most commonly used cell surface marker in GBM where it is shown to negatively correlate with patient outcome [11,36,37,38]. CD44 is another marker used to isolate a number of different CSCs lineages, such as colon, prostate, pancreatic and gastric cancer [4]. A number of studies also show that CD44 surface marker can be an indicator of patients’ poor survival in GBM [39,40].

Upon analysis of the TCGA-GBM dataset we observed a significant upregulation of both CD133 and CD44 in GBM patient tumours compared to non-tumour tissue. Additionally we highlighted that both markers were more highly expressed in GBM compared to lower grade gliomas (Figure 1) [27,38]. To enable us to study the effects of CYC065 and THZ1 in targeting GSCs in recurrent GBM in particular, we cultured patient-derived GBM cells isolated from recurrent tumours in serum-free conditions in both 2-D (monolayer) and 3-D (gliomasphere) cultures. While both the 2-D and 3-D culturing systems allowed for the maintenance of the GSC biomarkers, CD133 and CD44, we selected to conduct our studies using the 3-D culturing system as such systems are more representative of the parental tumour, especially in terms of treatment resistance [41]. As further evidence of the suitability of our 3-D cultures to interrogate novel treatment options to target GSCs, we observed, using Light Sheet Fluorescence Microscopy (LSFM) that the localisation of the CD133-positive and CD44-positive cells in each gliomasphere culture was unique and presumably reflected the different genetic background of the original tumours. Additionally, and while not studied specifically, we hypothesise that the higher expression of GSC CD133-postive cells in the sphere core is due to the hypoxia-like environment found in the core which usually leads to increased stemness [42]. Moreover, CD133 and CD44 GSC biomarker-negative cells were converted into GSC biomarker-positive cells after 12 days of culture in serum-free conditions, further contributing to the enrichment in GSCs within the sphere cultures. A similar result was found in rodent models using CD133-negative cells [10]. This highlights the plasticity of GSC biomarker-negative cells which could also potentially contribute to be the cause for the observed resistance to the conventional therapies in GBM.

We applied two transcriptional inhibitors, CYC065 and THZ1 to target CD133 and CD44 double- or single-positive GSCs in recurrent gliomaspheres as well as double-negative subpopulations. Since GSCs are known to effectively resist standard chemotherapy, we also studied TMZ as a monotherapy or in combination with the CDK inhibitors. Resistance to DNA alkylating agents in GSCs can be acquired through the regulation of the cell cycle which is slower in GSCs during chemotherapy treatment causing cells entering the quiescent state [43]. However, once chemotherapy is withdrawn dormant stem cells can resume tumourigenesis and in that way escape genotoxicity induced apoptosis [44]. As expected, TMZ was inefficient in targeting GSCs in the gliomaspheres [45] while both transcriptional CDK inhibitors, CYC065 and THZ1 were highly effective in reducing sphere size, and inducing apoptotic cell death in all isolated stem cell subpopulations in both recurrent cultures. From previous work, we propose that the observed apoptotic death of the GSCs was facilitated by CKI-induced downregulation of anti-apoptotic proteins, especially Mcl-1 [19,46]. The internal expression of CD133-positive cells within the spheres and decrease in marker expression upon CYC065 and THZ1 treatment supports the hypothesis that these CKIs successfully penetrate the spheres.

Overall, the high anti-cancer activity of both CYC065 and THZ1 in recurrent GSC cultures highlights the potential of these CDK inhibitors as an alternative to overcome treatment resistance to conventional therapies in GBM. Further investigations using preclinical animal models are needed to validate these in vitro findings. 

## Figures and Tables

**Table 1:** Patient clinical characteristics.

Cell Line	Sex	Age at Diagnosis	Diagnosis	Alterations	MGMT Status
GTCC-9	M	44.00	Recurrent	EGFR amp, PTEN loss	NA
GTCC-10	M	50.38	Recurrent	NA	Methylated

MGMT = O-6-methylguanine-DNA methyltransferase; M = male; EGFR = epidermal growth factor receptor; amp = amplification; PTEN = phosphatase and tensin homolog; NA = not available.

## Data Availability

Publicly available datasets were analyzed in this study. This data can be found here: http://gliovis.bioinfo.cnio.es/ (accessed on 23 April 2021).

## Supplementary Materials

The following are available online at https://www.mdpi.com/article/10.3390/cells10051182/s1, Figure S1: Gate settings for the CD133-biomarker positive and CD44-biomarker positive GSCs (FACS analysis); Figure S2: CD133 and CD44 mRNA expression in different subtypes of GBM; Figure S3: Genetic depletion of CDK2/9 or 7 cause viability loss in recurrent gliomaspheres; Figure S4: Gate settings for the CD133-biomarker positive and CD44-biomarker positive GSCs; Figure S5: CYC065 and THZ1 induce apoptotic cell death in CD133 and CD44 GSC biomarker-negative subpopulations and CD133 and CD44 GSC biomarker-positive subpopulations in recurrent GTCC-10 gliomaspheres.
