# Supplementary material for: Transcriptional CDK Inhibitors CYC065 and THZ1 Induce Apoptosis in Glioma Stem Cells Derived from Recurrent GBM
Source: Cells. 2021 May 12;10(5):1182. doi: 10.3390/cells10051182 (PMC8151379; doi:10.3390/cells10051182)
Supplement: Supplementary file 1 [file cells-10-01182-s001.zip › cells-1185824-supplementary.pdf]

cells MDPI

Supplemental Material for

Transcriptional CDK inhibitors CYC065 and THZ1 induce apoptosis in glioma stem cells derived from recurrent GBM

By

Viktorija Juric [1], Heiko Düssmann [1,2], Martine L.M. Lamfers [3], Jochen H.M. Prehn [1,2], Markus Rehm [4,5], Brona M. Murphy [1,*]

* Correspondence: bronamurphy@rcsi.com (B.M.M.)

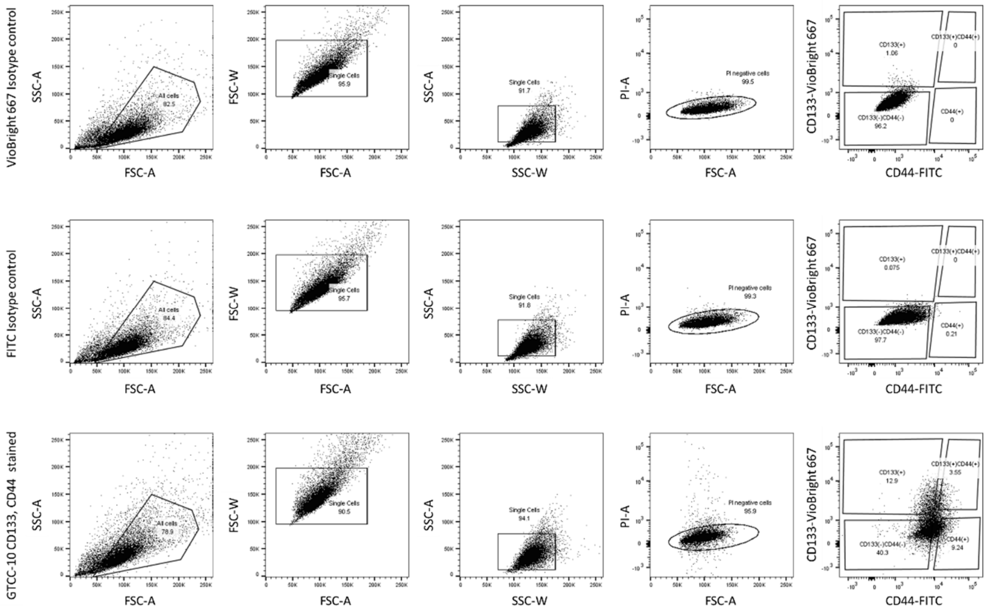

Figure S1. Gate settings for the CD133-biomarker positive and CD44-biomarker positive GSCs (FACS analysis). IgG1-VioBright 667 (upper panel) and IgG1-FITC (middle panel) used to set up the gates for CD133-positive and CD44-positive sub-populations in GTCC-10 cells. GTCC-10 cells stained with anti-CD133-VioBright 667 and anti-CD44-FITC markers (lower panel).

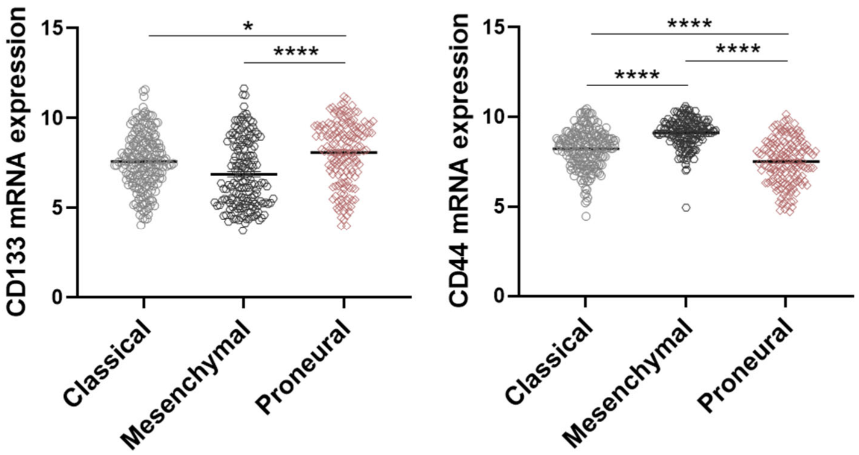

Figure S2. CD133 and CD44 mRNA expression in different subtypes of GBM. TCGA-GBM dataset was used to compare the expression of CD133 (left panel) and CD44 (right panel) between different subtypes of GBM. One-way ANOVA with post-hoc Tukey's analysis was used for statistical analysis, whereby, *<0.05, ****p<0.0001.

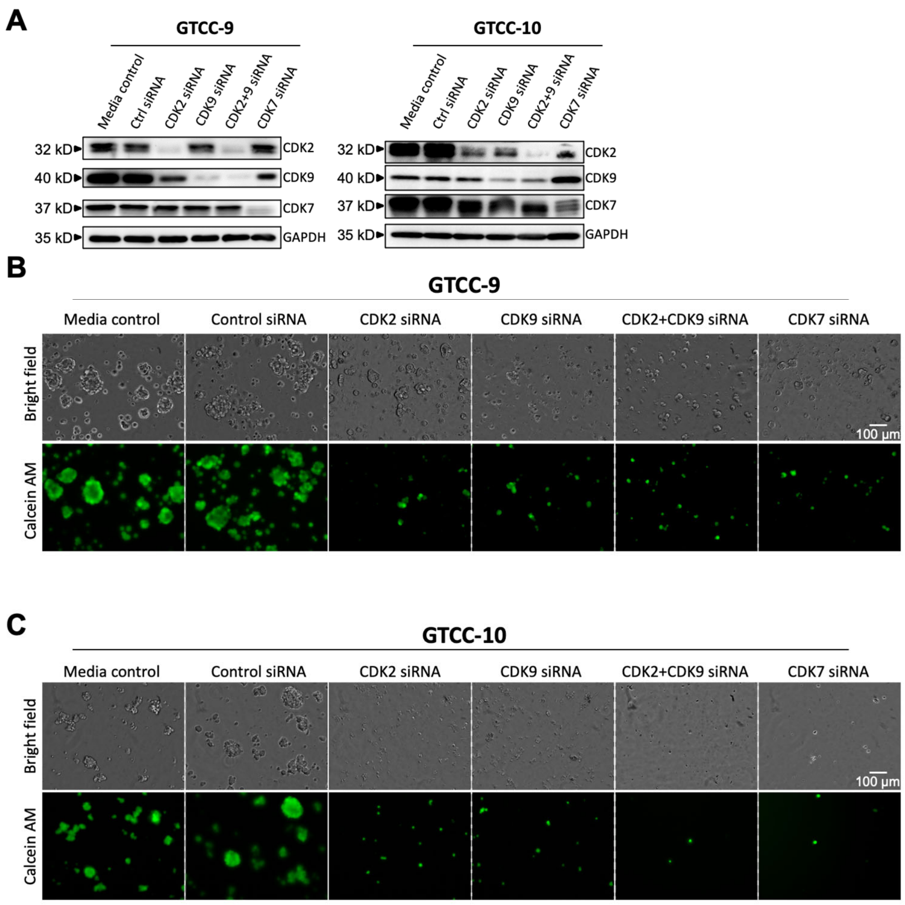

Figure S3. Genetic depletion of CDK2/9 or 7 cause viability loss in recurrent gliomaspheres. (**A**) Western blot analysis of GTCC-9 (left panel) and GTCC-10 (right panel) gliomaspheres were transfected with scrambled control siRNA (20 nM), Cdk2 and/or 9, 7-targeting siRNAs (20 nM) (as indicated). GAPDH was used as a loading control. Western blot analysis was performed in n=3 biological replicates and representative blots are shown here. (**B, C**) Morphological features and Calcein-AM staining of gliomaspheres with indicated genetic depletions. Images were taken with a Nikon Eclipse TE 300 inverted microscope, equipped with an 20x/0.45 NA objective and the appropriate filter cube for Calcein-AM (scale bar = 100 µm). N=3 independent experiments performed in triplicate for each condition.

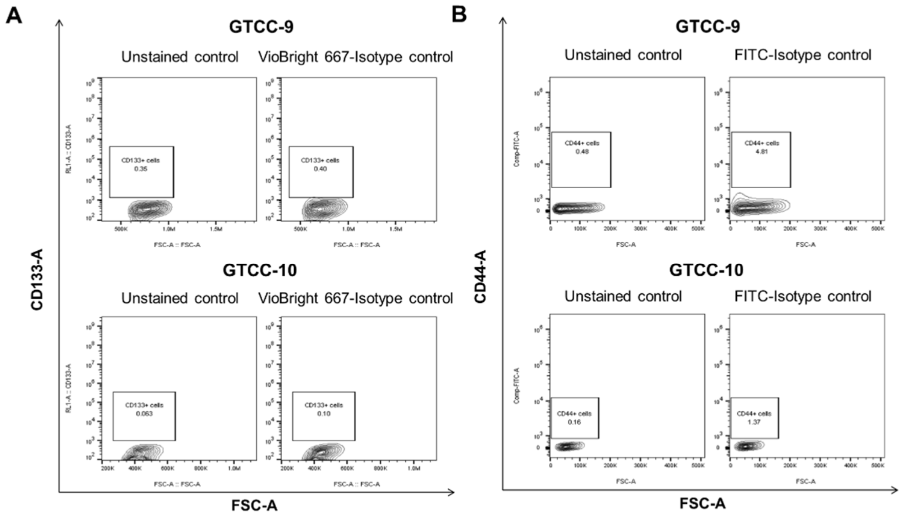

Figure S4. Gate settings for the CD133-biomarker positive and CD44-biomarker positive GSCs. (**A**,**B**) Unstained cells and corresponding isotype control stained cells were used to set up the gates for CD133- and CD44-positive cells in Figure 5.

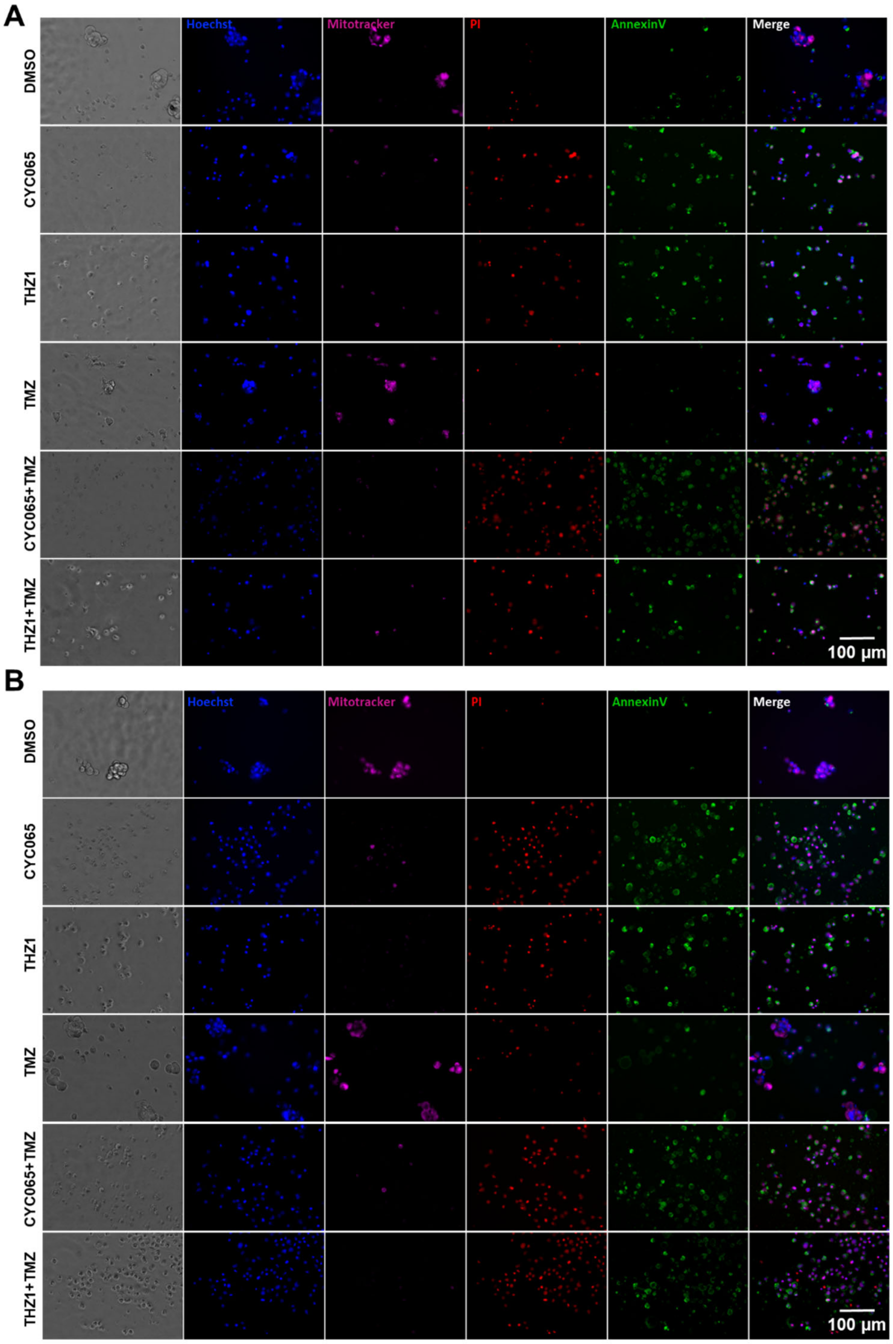

Figure S5. CYC065 and THZ1 induce apoptotic cell death in CD133 and CD44 GSC biomarker-negative subpopulations and CD133 and CD44 GSC biomarker-positive subpopulations in recurrent GTCC-10 gliomaspheres. (**A**) CD133, CD44-negative subpopulation of GTCC-10 gliomaspheres and (**B**) CD133, CD44-positive subpopulation of GTCC-10 gliomaspheres. Sorted subpopulations were treated with DMSO, 150 µM TMZ, 3 µM CYC065 or 100 nM THZ1 alone or pre-treated with TMZ (150 µM, 24 h) and imaged 120 h after treatment. Scale bar = 100 µm; n=3 independent experiments were performed.
